# FACTORS ASSOCIATED WITH LONG-TERM FUNCTIONAL OUTCOMES AND PARTICIPATION IN PATIENTS WITH COLORECTAL CANCERS

**DOI:** 10.2340/jrm.v58.43262

**Published:** 2026-01-07

**Authors:** Bhasker AMATYA, Helen MOHAN, Krystal SONG, Alexander HERIOT, Fary KHAN

**Affiliations:** 1 Department of Rehabilitation Medicine, Peter MacCallum Cancer Centre and Royal Melbourne Hospital, Parkville, Victoria; 2 Department of Medicine (Royal Melbourne Hospital), University of Melbourne, Parkville, Victoria; 3 Australian Rehabilitation Research Centre, Royal Melbourne Hospital, Parkville, Victoria; 4 Department of Colorectal Surgery, Peter MacCallum Cancer Centre, Melbourne, Victoria, Australia

**Keywords:** colorectal cancer, rehabilitation, impairment, function, quality of life

## Abstract

**Objective:**

To assess the longer-term functional, psychosocial, and participation outcomes in colorectal cancer (CRC) survivors.

**Methods:**

Adult CRC survivors (*n* = 100) living in the community completed validated questionnaires. Descriptive statistics summarized participant characteristics, while multivariate linear regression (*p* < 0.05) identified predictors of functional and psychosocial outcomes, and a binary logistic regression model was applied to identify factors associated with poorer QoL.

**Results:**

Participants (mean age: 62.2 ± 12.7 years, 53% male, 64% with grade III–IV CRC, mean time since diagnosis of 2.4 ± 1.6 years), reported persistent challenges, including fatigue (77%), bowel dysfunction (50%), sleep disturbances (49%), fear of recurrence (48%), and pain (42%). Despite this, functional recovery was good (Clinical Functioning Information Tool (Mean): 50.3 ± 42.4), with minimal mental health impact (Depression Anxiety Stress Scale: 11.3 ± 17.2). CRC-specific quality of life was poor (Functional Assessment of Cancer Therapy-Colorectal: 97.4 ± 14.5). Community integration was fair (Community Integration Questionnaire: 18.4 ± 6.5), with moderate satisfaction in overall health (Euro Quality of Life: 71.5 ± 18.6). Regression analysis identified age > 60 years, female gender, fatigue, pain, radiotherapy, and time since diagnosis > 3 years as significant predictors of poorer outcomes.

**Conclusion:**

Persistent challenges faced by CRC survivors underscore the need for personalized, interdisciplinary rehabilitation-inclusive survivorship care addressing ongoing disabilities, psychosocial issues, and unmet needs.

## LAY ABSTRACT

Colorectal cancer (CRC) survivors can face long-term challenges after completing treatment. This study looked at the experiences of 100 Australian CRC survivors living in the community. Many participants reported ongoing issues such as fatigue, bowel problems, sleep disturbances, and fear of cancer returning. Despite these difficulties, most regained good physical function and maintained stable mental health. However, their overall quality of life remained low, and their ability to reintegrate into the community was only moderate. Factors like older age, being female, fatigue, and pain made recovery more difficult. These findings highlight the need for a more personalized and team-based approach to rehabilitation-inclusive survivorship care, which addresses not just physical recovery but also emotional and social well-being to improve long-term outcomes for CRC survivors.

Colorectal cancer (CRC) is the third most common cancer, representing 10% of all cancers, with over 1.9 million new cases diagnosed globally in 2020 alone (1). It is the second leading cause of cancer mortality, with 9.4% of all cancer deaths worldwide (over 935,000 deaths) in 2020 (1). The global burden of CRC continues to rise and is projected to reach approximately 3.2 million new cases and 1.6 million deaths annually by 2040 (2). Incidence and mortality rates vary significantly, with higher rates observed in males, older adults (65–75 years), and in low- and middle-income countries (2, 3). The global economic burden of CRC remains unknown; however, it is resource-intensive and associated with a significant financial burden for patients/families and the healthcare system (4).

Therapeutic advancements have prolonged the age-standardized global 5-year net survival range from 62% to 78% (5). However, CRC survivors frequently experience long-term sequelae, including functional, neurological, and psychosocial impairments, which limit everyday activity and participation. Postoperative complications such as gastrointestinal symptoms (such as excessive flatulence, abdominal wall issues, pain, bloating, constipation/diarrhoea, faecal incontinence, nausea. etc.), stoma-related challenges (such as body image and psychosocial issues), and chemotherapy-induced side effects (such as neuropathy, fatigue, immune suppression, cardiotoxicity, cachexia), are prevalent (6, 7). Further, survivors often face psychological issues, including depression (57%) and anxiety (42%), compounded by the distress of a cancer diagnosis (8). Further, in the transitional period, various adjustment issues, such as increased care needs, inability to drive and return to work, financial constraints, marital stress, and limitations in societal participation may surface (9). These challenges significantly impact patients quality of life (QoL), contributing to prolonged hospital stays, higher readmission rates, and increased healthcare costs (9).

A population-based Australian study quantifying physical and mental health-related outcomes in people with 13 various cancer types (*n* = 22,505 participants, 13% CRC survivors) reported that compared with people without cancer, cancer patients had a greater disability, psychological and poor/fair QoL (10). One longitudinal study (*n* = 539) reported that at 6–12 months post-CRC diagnosis, most patients reported difficulties in everyday usual activities (26%), pain and/or discomfort (25%), anxiety and/or depression (23%), and mobility issues (15%) (11). Further, pain or discomfort escalated over time (11). Longitudinal studies show QoL trajectories vary, with some survivors reporting consistently poor QoL or a decline over the years (12). One systematic review (*n* = 8 studies) evaluated prognostic factors for return to work and work disability in CRC survivors and found (neo) adjuvant therapy, higher age, and more comorbidities had a significant negative influence on return to work (13). Moreover, unmet supportive care needs range from psychological and physical assistance to prevalent information gaps, particularly among younger survivors, those with lower socioeconomic status, and/or those undergoing extensive treatment (14).

The overriding objective of cancer care has shifted beyond survival to the holistic reintegration of survivors into their communities. This transition necessitates a focus on the longer-term management of CRC survivors, addressing complex and multifaceted rehabilitation needs (social reintegration, return to driving/work, and psychosocial outcomes). However, research on functional outcomes and “needs” assessment is limited. This study aims to evaluate factors associated with residual disability, functional outcomes, psychosocial sequelae, and unmet care needs in CRC survivors within an Australian community cohort. These will provide insights into clinical and research priorities and contribute to improved survivorship care.

## METHODS

### Study design

A prospective observational study was conducted using the Strengthening the Reporting of Observational Studies in Epidemiology (STROBE) criteria (15) and approved by the Institutional Research and Ethics Committee (HREC/100818/PMCC).

### Participants and setting

This study was conducted at the Peter MacCallum Cancer Centre (PMCC), a tertiary referral center in Victoria, Australia. The sample was drawn from patients discharged post-surgery since January 2017 and residing in the community. Participant selection was facilitated through cross-referencing the PMCC Electronic Medical Record system and the CRC patient database to ensure accuracy. The inclusion and exclusion criteria for participants are detailed in Box 1.

**Box 1. Participants’ inclusion and exclusion criteria**

**Table:** 

*Inclusion criteria*	*Exclusion criteria*
Aged 18 years and over Ability to consent Confirmed diagnosis (main) of CRC Underwent primary resection surgery for the CRC at the PMCC	Currently on any active CRC treatment Unable to communicate in English and unable to answer questionnaires Patients with significant comorbidities or medically unstable, or psychiatric disorders limiting participation in the study

### Procedure

*Data collection.* Based on the study selection criteria, a trained research assistant invited all eligible patients identified in the PMCC database to participate via telephone or email. A research officer followed up within a week to provide additional details concerning the study. Those who consented were recruited and interview appointments were scheduled based on availability and convenience. A trained research assistant conducted all interviews and assessments (approximately 30–35 min) using a structured format via telephone or online communication platforms (e.g., Skype, Zoom, MS Teams). Data collection used standardized instruments (see Measures). While assessors did not prompt participants, they assisted those having trouble with the questionnaires and offered appropriate rest breaks during the sessions.

### Measures

*Demographic and CRC-related information* was collected from the electronic medical records by the clinician and included sociodemographic data and clinical characteristics data. The International Classification of Functioning, Disability and Health (ICF) (16) was used as a conceptual basis for the choice of the best outcomes for measurement.

*Clinical Functioning Information Tool (ClinFIT)* (17) is an ICF-based 30-item tool assessed “functioning” and disability: 9 items in the “*Body Functions*” domain, and 21 items in the “*Activities and Participation*” domain. Clinicians used these categories, defined by clinically meaningful descriptions, to assess patient functioning on an 11-point numeric rating scale (0 = no problem to 10 = complete problem). It also includes the ICF Generic-7 Set, consisting of 7 categories, which provides a minimum set of information on functioning that can be collected across various health conditions and clinical settings.

*Depression Anxiety Stress Scale- 21 (DASS)* (18) comprises 3 x 7-item self-report scales, to measure the negative emotional states of depression, anxiety, and stress. Participants rated the extent to which they experienced each state over the past week on a 4-point Likert scale, with higher scores indicating more dysfunction (18).

*European Quality of Life Scale (EQ-5D-5L)* (19) assessed overall QoL in 5 health dimensions: mobility, self-care, daily activity, pain/discomfort, and anxiety/depression, with responses on 5 ordinal levels: no problems (0) to extreme problems (4). The sixth item within the scale assessed participants’ current overall health using a visual analogue scale (VAS) from 0 (worst health state) to 100 (best health state).

*Functional Assessment of Cancer Therapy-Colorectal (FACT-C) scale (**20**)* assessed the health-related QoL in the following domains: physical, functional, social/family, emotional well-being, and colorectal cancer-specific. The participants rated the degree to which a given item applied to them during the past 7 days using a 5-point Likert scale (0 = not at all to 4 = very much).

*Community Integration Measure-Revised (CIQ-R) (**21**)* assessed community participation across 4 subscales: home integration, social integration, productivity, and electronic social networking.

*Cancer Survivor Unmet Needs Measure (CaSUN)* (22) assessed the “needs” experienced by CRC survivors within the preceding month in 5 domains: existential survivorship, comprehensive care, information, QoL and relationships, and 6 positive change items. Need items were scored no need (0), “met” need (1) or “unmet” need (2); “unmet” needs were further rated as weak, moderate, or strong. The sum of responses was used to calculate the total unmet and total need.

### Data analysis and statistics

Descriptive statistics were used to summarize participant characteristics, with continuous variables presented as mean ± standard deviation (SD) and categorical variables as frequencies and percentages. Univariate analyses explored associations between independent variables (demographic and clinical characteristics) and outcome measures. For continuous outcome variables, independent *t*-tests were used to compare mean differences between groups, while χ^2^ tests examined categorical variables. Further, multiple linear regression investigated the independent associations between potential sociodemographic and clinical variables of interest (e.g., variables with significant associations or those with theoretical importance) with outcome measure scores. Prior to modelling, assumptions of linearity, normality of residuals, homoscedasticity, and absence of multicollinearity were checked. A backward selection method was applied, starting with all candidate variables in the model. Variables are removed one at a time, starting with a significance threshold of *p* < 0.05. The independent variables were categorized as follows: age and time since diagnosis as continuous variables; CRC type: as colon (reference) vs rectal; age at diagnosis ≤ 60 (reference) vs > 60 years, time since diagnosis ≤ 3 (reference) vs > 3 years, treatment type: chemotherapy or radiotherapy (reference) vs none, comorbidity: present (reference) vs absent; marital status: married (reference) vs single/divorced; employment: employed (reference) vs unemployed/retired/student; symptoms: recurring pain or fatigue (reference) vs no symptoms; and rehabilitation: inpatient or community rehabilitation (reference) vs none. A *post hoc* analysis using the Bonferroni adjustment was applied to between-group comparisons to reduce the likelihood of Type I errors. The alpha level (0.05) was divided by the number of covariates/tests to account for multiple comparisons. This approach aligned with the study’s descriptive nature, ensuring that all potentially important predictors of short- and long-term sequelae of CRC were identified. Further, a binary logistic regression analysis was conducted to identify factors associated with poorer QoL, as measured by the FACT-C total score (dependent variable), which was dichotomized using a predefined cut-off of the sample mean (≤ 97.4 = good QoL, > 97.4 = poorer QoL). The backward stepwise method was applied to retain only significant predictors, with odds ratios (OR) and 95% confidence intervals (CI) reported. For the CaSUN measure, all 35 items were included in calculating the total “met”, total “unmet”, and total needs for each domain. All statistical analyses were performed using IBM SPSS for Windows, version 22.0 (IBM Corp, Armonk, NY, USA), with statistical significance set at *p* < 0.05.

## RESULTS

### Sample characteristics

The sociodemographic and clinical characteristics of the sample are presented in Table I.

**Table I:** Sociodemographic and clinical characteristics of participants (*n* = 100)

Characteristics	Statistics
Age, years, mean ± SD, range	62.2 ± 12.7, 24.6–83.2
Sex: Male, *n* (%)	53 (53.0)
Caucasian, *n* (%)	93 (93.0)
Marital status (Pre-diagnosis), *n* (%)	
Married/Partner	70 (70.0)
Single/Divorced/Separated	30 (30.0)
Marital status (Post-diagnosis), *n* (%)	
Married/Partner	58 (58.0)
Single/Divorced/Separated	42 (42.0)
Living with (Pre-diagnosis), *n* (%)	
Alone	22 (22.0)
Partner/Family	77 (77.0)
Living with (Post-diagnosis), *n* (%)	
Alone	31 (31.0)
Partner/Family	67 (67.0)
Education, *n* (%)	
Secondary	72 (72.0)
Tertiary	26 (26.0)
Employment (Pre-diagnosis), *n* (%)	
Full-time	46 (46.0)
Part-time	6 (6.0)
Unemployed/retired	40 (40.0)
Employment (Post-diagnosis), *n* (%)	
Full-time	27 (27.0)
Part-time	7 (7.0)
Unemployed/retired	52 (52.0)
Smokers, *n* (%)	54 (54.0)
Consumes alcohol, *n* (%)	79 (79.0)
Private health insurance, *n* (%)	25 (25)
Disease duration, years, mean ± SD, range	2.5 ± 1.6, 1.0–7.2
Age at diagnosis, years, mean ± SD, range	59.7 ± 1.6, 20.8–82.5
Family history, *n* (%)	40 (40.0)
CRC Type	
Colon	45 (45.0)
Rectum	55 (55.0)
CRC stage at diagnosis (*n* = 77), *n* (%)	
Stage I–II	28 (36.3)
Stage III–IV	49 (63.7)
Surgery type, *n* (%)	72 (67.9)
Open	37 (37.0)
Robotic	49 (49.0)
Other	10 (10.0)
Surgery side effects (*n* = 96), *n* (%)	52 (54.2)
Mild	19 (19.8)
Severe	17 (17.7)
Stoma, *n* (%)	
Present	36 (36.0)
Reserved	25 (25.0)
Chemotherapy, *n* (%)	73 (73.0)
Side effects (*n* = 67)	56 (82.1)
Severe side effects	16 (23.9)
Radiotherapy, *n* (%)	49 (49.0)
Side effects (*n* = 40)	25 (62.5)
Severe side effects	5 (12.5)
CRC recurrence, *n* (%)	30 (30.0)
Remission, *n* (%)	53 (53.0)
Co-morbidities, *n* (%)	86 (86.0)
Hypertension	43 (50.0)
Diabetes	13 (15.1)
Depression	15 (17.4)
IHD	12 (14.0)
Motor impairments, *n* (%)	4 (4.0)
Cognitive impairment, *n* (%)	17 (17.0)
Fear of recurrence, *n* (%)	48 (48.0)
Sleep problems, *n* (%)	49 (49.0)
Fatigue, *n* (%)	77 (77.0)
Sexual issues, *n* (%)	15 (11.6)
Pelvic floor problem, *n* (%)	17 (17.0)
Nausea, *n* (%)	12 (12.0)
Weight loss, *n* (%)	29 (29.0)
Skin issues, *n* (%)	23 (23.0)
Bladder dysfunction, *n* (%)	36 (36.0)
Bowel dysfunction, *n* (%)	50 (50.0)
Pain, *n* (%)	42 (42.0)
Pain score (0 no pain; 10 = extreme pain), mean ± SD, range	1.6 ± 2.4, 0–10
Inpatient rehabilitation, *n* (%)	75 (75.0)
CBR, *n* (%)	63 (63.0)

CRC: colorectal cancer; CBR: community-based rehabilitation; IHD: ischaemic heart disease; *n*: total number; SD: standard deviation.

Of the 540 patients in the database, a total of 100 eligible participants consented to participate in the study. The mean age of the participants was 62.2 ± 12.7 (range = 24.6–83.2) years; the majority were male (53%) and Caucasian (93%). The average time since CRC diagnosis was 2.5 ± 1.6 (range = 1.0–7.2) years, with a mean age at diagnosis of 59.7 ± 1.6 (range = 20.8–82.5) years. Most participants (86%) reported at least 1 comorbidity, including high blood pressure (43%), diabetes (15%), depression (15%), and ischaemic heart disease (12%). More than half (55%) had rectal cancer, and nearly half (49%) were diagnosed with advanced-stage (Grade III–IV). Forty-nine participants underwent robotic surgery, while 37 had open surgeries. Chemotherapy was received by 73% of participants, and 49% underwent radiotherapy. Over one-third (36%) currently have a stoma, 53% reported being in remission, whereas 30 participants experienced disease relapse.

### Participant-reported symptoms/impairments

The most frequently reported symptoms were fatigue (77%), sleep disturbances (49%), and fear of recurrence (48%). Forty-one participants reported CRC-related pain (mean pain score = 1.6 ± 2.4 on a 0–10 VAS) and half reported bowel dysfunction (50%). Bladder issues were noted by over one-third of the participants (36%), which was higher than anticipated. Other reported impairments included weight loss (29%), skin problems (23%), cognitive difficulties (17%), pelvic floor issues (17%), sexual dysfunction (15%), and nausea (12%) (see Table I).

### Current level of functioning, participation, psychological well-being, and QoL

Participants reported minimal changes in physical function, as reflected by low ClinFIT total scores (mean ± standard deviation [M ± SD] = 50.3 ± 42.4). Similarly, minimal changes were observed in negative emotional states, with low DASS-21 scores: total (11.3 ± 17.2), depression (6.0 ± 8.7), anxiety (1.3 ± 3.9), and stress (4.1 ± 6.9). Most participants reported good CRC-specific QoL, as measured by the FACT-C (total score = 97.4 ± 18.6) and the EQ-5D-5L overall health score (71.5 ± 18.6). Among the FACT-C subscales, the highest mean scores, indicating the most significant challenges, were observed in the social (22.8 ± 5.2) and physical (22.7 ± 5.5) well-being domains.

### Social and community reintegration aspect

At the time of assessment, employment rates declined notably compared with pre-diagnosis levels (34% vs 52%). Full-time employment also decreased substantially (27% vs 46%). Additionally, 52% of participants were either unemployed or had retired by the time of assessment. Marital or partnership status also showed some decline, with 67% of participants currently married or living with a partner, compared with 77% before their CRC diagnosis. Despite these factors, participants reported a positive adjustment to community living (CIQ-R total M ± SD = 18.4 ± 6.5). These findings highlight both the socioeconomic impact of CRC and the resilience of individuals in adapting to life post-diagnosis (Table II).

**Table II:** Descriptive statistics for subscales (*n* = 100)

Scales	Statistics Mean±SD (range)
ClinFIT (Total) (0–300)	50.3 ± 42.4 (0–175)
ClinFIT– Generic 7 total (0–70)	15.5 ± 13.7 (0–53)
DASS Total (0–126)	11.3 ± 17.2 (0–86)
Depression (0–42)	6.0 ± 8.7 (0–40)
Anxiety (0–42)	1.3 ± 3.9 (0–26)
Stress (0–42)	4.1 ± 6.9 (0–32)
EQ-5D-5L	
Mobility (0–4)	0.5 ± 0.8 (0–4)
Self-care (0–4)	0.3 ± 0.5 (0–2)
Daily activity (0–4)	1.0 ± 1.0 (0–4)
Pain/discomfort (0–4)	0.7 ± 0.9 (0–4)
Anxiety/depression (0–4)	0.7 ± 0.9 (0–3)
Overall health (0–100)	71.5 ± 18.6 (15–100)
FACT-C (0–168)	97.4 ± 14.5 (54–116)
Physical well-being (0–28)	22.7 ± 5.5 (2–28)
Social well-being (0–28)	22.8 ± 5.2 (4.7–28)
Emotional well-being (0–24)	19.5 ± 4.2 (4–24)
Functional well-being (0–28)	19.0 ± 6.2 (1–28)
Additional CRC concerns (0–60)	13.5 ± 3.8 (7–26)
TOI (0–116)	55.1 ± 8.4 (25–64)
FACT-G Total (0–108)	84.0 ± 17.3 (34.5–108)
CIQ-R Total (0–72)	18.4 ± 6.5 (2–31)
Home integration (0–12)	6.9 ± 3.4 (0–12)
Social integration (0–10)	5.7 ± 2.0 (1–10)
Productivity (0–7)	3.0 ± 1.6 (0–7)
Electronic social networking (0–6)	2.9 ± 1.6 (0–6)
CaSUN Total needs	13.9 ± 6.0 (5–35)
Total “unmet” needs	5.1 ± 6.9 (0–35)
Total “met” needs	8.8 ± 4.0 (0–21)

CASUN: Cancer Survivor Unmet Needs Measure; CIQ-R: Community Integration Quessionaire-Revised, ClinFIT: Clinical Functioning Infoirmation Tool; DASS: Depression Anxiety Stress Scale, EQ-5D-5L: Euro-Quality of Life scale; FACT-G: Functional Assessment of Cancer Therapy – General, FACT-C: Functional Assessment of Cancer Therapy – Colorectal; *n*: total number, SD: standard deviation, TOI: Trial Outcome Index.

### Supportive care needs and positive outcomes

All participants endorsed at least 1 need (“met” or “unmet”) in the CaSUN assessment. Three-quarters of the participants (75%) reported at least one “unmet” need, with an average of 5.1 ± 6.8 (range = 0–6.8). The most endorsed total needs (“met” and “unmet”) were in the domains of existential survivorship (M ± SD = 4.35 ± 3.2) and comprehensive cancer care (4.07 ± 0.8). The most frequently reported “unmet” needs were also within the “existential survivorship” domain (0.9 ± 1.0), followed by the “comprehensive cancer care” domain (0.5 ± 0.8). The highest reported “unmet” needs included reducing stress in life, managing ongoing side effects and complications, adjusting to body image, and obtaining information on support and benefits. Participants expressed satisfaction (“met” needs) with the care and information they received, as well as with emotional and medical support: “comprehensive cancer care” domain (3.2 ± 1.0) (Table III).

**Table III:** Participants’ endorsement of the Cancer Survivor Unmet Needs (CaSUN) Measure ranked by total “met” needs (*n* = 100)

Rank	CaSUN factor	Total met need		Total unmet need	
		Mean (SD)	Range	Mean (SD)	Range
1	Comprehensive cancer care	3.2 (1.2)	0–5	0.5 (0.8)	0–3
2	Existential survivorship	2.3 (2.0)	0–9	0.9 (1.0)	0–3
3	Information	2.3 (1.1)	0–3	0.2 (0.5)	0–2
4	Relationship	0.2 (0.4)	0–2	0.2 (0.4)	0–2
5	Quality of life	0.4 (0.5)	0–2	0.4 (0.6)	0–2

CaSUN: Cancer Survivor Unmet Needs measure, *n*: total number, SD: standard deviation.

### Factors associated with the current level of functioning and well-being

Table IV presents the results of univariate analysis, showing the impact of demographic and clinical covariates, and Table V presents the results of multivariate linear regression, with β coefficients indicating the strength and direction of associations between the independent variables and the outcome measures. The findings are summarized below:

**Table IV:** Univariate statistics for the impact of demographic and clinical variables associated with the outcome measures

Outcome measures^†^	Demographic and clinical characteristics variables^§^													
	Age	Sex	Marital status	Employment	Comorbidity	Age at diagnosis	CRC type	CRC duration	Stoma	CT	RT	Pain	Fatigue	IPR
ClinFIT														
Total	*0.03*	0.11	0.51	*0.008*	*0.035*	0.32	0.14	0.26	0.38	0.41	**0.003**	**< 0.001**	**< 0.001**	0.77
Generic 7	0.15	0.06	0.36	*0.005*	0.10	0.87	0.72	0.22	0.37	0.28	*0.008*	**< 0.001**	**< 0.001**	0.56
DASS														
Total	0.51	0.10	0.16	0.053	0.46	0.70	0.47	0.27	0.61	0.62	0.17	*0.01*	**0.003**	0.19
Depression	0.43	0.13	0.12	0.09	0.87	0.70	0.50	0.66	0.80	0.58	0.12	*0.009*	**0.002**	0.40
Anxiety	0.45	0.26	0.35	*0.04*	0.27	0.65	0.78	0.24	0.30	0.89	0.40	0.16	0.10	0.56
Stress	0.83	0.80	0.31	0.12	0.32	0.83	0.42	0.12	0.70	0.65	0.33	*0.04*	*0.008*	0.06
EQ-5D-5L														
Health	0.99	0.17	*0.04*	0.84	0.94	0.91	0.70	0.82	0.81	0.66	0.63	*0.02*	**< 0.001**	0.49
Mobility	0.42	0.54	0.97	0.06	0.16	0.82	0.26	0.10	0.67	0.18	**0.001**	*0.04*	0.81	*0.02*
Self-care	0.11	0.63	0.71	**0.004**	0.56	0.44	0.50	0.53	0.73	0.98	0.16	*0.01*	0.35	0.50
Daily activity	0.46	0.16	0.49	**0.003**	0.06	0.86	0.09	0.16	0.12	0.07	**< 0.001**	**< 0.001**	**< 0.001**	0.24
Pain/discomfort	0.67	0.70	0.67	0.46	0.30	0.67	0.41	*0.04*	0.35	0.46	0.75	**< 0.001**	0.07	0.90
Anxiety/depression	0.10	*0.04*	0.07	0.16	0.09	0.89	0.34	0.18	0.77	0.44	0.06	0.08	*0.007*	0.50
FACT-C														
Total	0.13	0.49	*0.02*	*0.007*	0.42	0.55	0.98	0.67	0.96	0.39	0.16	**< 0.001**	**< 0.001**	0.89
Physical wellbeing	0.89	0.48	0.48	*0.04*	0.44	0.36	0.83	0.68	0.61	0.26	0.08	**< 0.001**	**< 0.001**	0.94
Social well-being	*0.02*	0.67	**< 0.001**	0.28	0.47	*0.01*	0.70	0.45	0.30	0.77	0.91	0.06	0.11	0.61
Emotional well-being	0.24	0.14	0.09	*0.03*	0.68	0.89	0.59	0.10	0.68	0.81	0.16	*0.02*	**0.001**	0.57
Functional well-being	0.13	0.40	0.27	*0.006*	0.72	0.85	0.30	0.55	0.47	0.10	*0.008*	**< 0.001**	**< 0.001**	0.09
CRC concerns	0.30	0.45	0.46	0.26	0.10	0.92	*0.05*	0.32	0.43	0.10	**< 0.001**	*0.03*	**< 0.001**	*0.02*
TOI	0.57	0.46	0.34	**0.004**	0.45	0.69	1.00	0.79	0.61	0.23	0.10	**< 0.001**	**< 0.001**	0.79
FACT-G														
Total	0.13	0.45	*0.03*	*0.012*	0.40	0.63	0.65	0.89	0.83	0.27	0.06	**< 0.001**	**< 0.001**	0.54
CIQ-R														
Total	**< 0.001**	*0.02*	**< 0.001**	**< 0.001**	0.10	**< 0.001**	0.43	0.42	*0.04*	0.91	*0.02*	0.58	*0.007*	0.10
Home integration	0.07	**0.003**	**< 0.001**	0.10	0.69	0.21	0.42	0.26	*0.02*	0.97	*0.02*	0.81	0.32	0.91
Social integration	**0.002**	0.39	0.84	022	0.92	0.08	0.63	0.66	0.17	0.34	*0.01*	0.17	*0.03*	0.66
Productivity	**< 0.001**	0.80	0.39	**< 0.001**	0.10	**< 0.001**	0.87	0.10	0.84	0.36	0.90	0.055	**< 0.001**	0.65
E-social network	**< 0.001**	*0.005*	0.19	0.33	0.32	**< 0.001**	0.54	0.76	*0.04*	0.73	008	0.15	0.10	0.33

* Values significant after Bonferroni adjustment (set at 0.05/14 tests) *p* < 0.004 are shown in bold and those significant at 0.05 level are italicized.

§ Variables references: Gender: male vs female; Age groups: ≤ 60 vs >60 years; Marital status: married vs single; Employment status: employed vs unemployed; Comorbidity: present vs none; CRC type: colon vs rectal; CRC duration: ≤3 vs >3 years; Age at diagnosis: ≤ 60 vs > 60 years; Stoma: none or yes (present/reversed); Chemotherapy (CT): received vs none; Radiotherapy (RT): received vs none; Pain: present vs none; Fatigue: present vs none; Inpatient rehabilitation (IPR): received vs no.

CIQ-R: Community Integration Questionnaire-Revised, ClinFIT: Clinical Functioning Information Tool, CRC: colorectal cancer, CT: chemotherapy, DASS: Depression Anxiety Stress Scale, EQ-5D-5L: Euro Quality of Life Scale; FACT-G: Functional Assessment of Cancer Therapy – General, FACT-C: Functional Assessment of Cancer Therapy – Colorectal cancer; TOI: Trial Outcome Index.

**Table V:** Multivariate regression model evaluating independent demographic and clinical variables for the outcome measures

Outcome measures^†^	Demographic and clinical characteristics variables^§^													
	Age	Sex	Marital status	Employment	Comorbidity	Age at diagnosis	CRC type	CRC duration	Stoma	CT	RT	Pain	Fatigue	IPR
ClinFIT														
Total	0.44*	NS	NS	NS	NS	–0.32*	NS	NS	NS	NS	**0.41^**	**0.34^**	**0.34^**	NS
Generic 7	0.32*	0.17*	NS	NS	NS	–0.35*	–0.23*	NS	NS	NS	**0.43^**	**0.35^**	**0.37^**	NS
DASS														
Total	NS	NS	NS	NS	NS	NS	NS	NS	–0.26*	NS	0.28*	NS	**0.30** ^**#**^	NS
Depression	NS	NS	NS	NS	NS	NS	NS	NS	NS	NS	NS	0.22*	**0.32** ^**#**^	NS
Anxiety	NS	NS	NS	NS	NS	NS	NS	NS	NS	NS	NS	NS	NS	NS
Stress	NS	0.22*	NS	NS	NS	NS	NS	–0.23*	NS	NS	NS	NS	0.25*	0.24*
EQ-5D-5L														
** **Health	NS	NS	NS	NS	NS	NS	NS	NS	NS	NS	NS	–0.22*	**–0.41^**	NS
Mobility	NS	NS	NS	NS	0.32*	NS	NS	NS	NS	NS	0.44*	NS	NS	NS
Self-care	NS	NS	NS	NS	NS	NS	NS	NS	NS	NS	NS	0.26*	NS	NS
Daily activity	NS	0.17*	NS	0.19*	NS	NS	NS	NS	NS	NS	**0.51^**	**0.33^**	**0.33^**	NS
Pain/discomfort	NS	NS	NS	NS	NS	NS	NS	–0.21*	NS	NS	NS	**0.62^**	0.22*	NS
Anxiety/depression	NS	**0.22** ^**#**^	NS	NS	NS	NS	NS	NS	NS	NS	NS	NS	0.25*	NS
FACT-C														
** **Total	NS	NS	–0.18*	NS	NS	NS	NS	NS	NS	NS	NS	**–0.37^**	**–0.37^**	NS
Physical well-being	NS	NS	NS	NS	NS	NS	NS	NS	NS	NS	–0.30*	**–0.47^**	**–0.35^**	NS
Social well-being	NS	NS	**–0.43^**	NS	NS	NS	NS	NS	NS	NS	NS	NS	NS	NS
Emotional well-being	NS	NS	NS	NS	NS	0.35*	NS	NS	NS	NS	NS	–0.20*	**–0.33** ^**#**^	NS
Functional well-being	–0.39*	NS	NS	NS	NS	0.37*	NS	NS	NS	NS	**–0.36** ^**#**^	**–0.26** ^**#**^	**–0.43^**	NS
CRC concerns	**0.45** ^**#**^	NS	NS	NS	NS	–0.35*	NS	NS	NS	NS	**0.38** ^**#**^	NS	**0.39^**	NS
TOI	NS	NS	NS	–0.25*	NS	0.29*	NS	NS	NS	NS	–0.29*	**–0.43^**	**–0.37^**	NS
FACT-G: Total	NS	NS	NS	NS	NS	NS	NS	NS	NS	NS	–0.30*	**–0.35^**	**–0.39^**	NS
CIQ-R														
** **Total	**–0.43** ^**#**^	NS	**0.38^**	NS	NS	NS	NS	NS	NS	NS	**–0.41^**	NS	**–0.23** ^**#**^	NS
Home integration	NS	NS	**0.63^**	NS	NS	NS	NS	0.17*	NS	NS	**–0.37^**	NS	NS	NS
Social integration	–0.46*	NS	NS	NS	NS	NS	NS	NS	NS	NS	–0.36*	NS	NS	NS
Productivity	**–0.39** ^**^**^	NS	NS	**–0.55^**	NS	NS	NS	NS	NS	NS	NS	–0.16*	**–0.25^**	NS
E-social network	**–0.51** ^**#**^	0.20*	NS	NS	NS	NS	NS	NS	NS	NS	–0.30*	NS	NS	NS

* *p* < 0.05,

# *p* < 0.01,

^ *p* < 0.001;

† standardized β coefficients; NS: not significant.

§ Variables references: Gender: male vs female; Age: years; Marital status: married vs single; Employment status: employed vs unemployed; Comorbidity: present vs none; CRC type: colon vs rectal; CRC duration: years; Age at diagnosis: years; Stoma: none vs yes (present/reversed); Chemotherapy (CT): received vs none; Radiotherapy (RT): received vs none; Pain: present vs none; Fatigue: present vs none; Inpatient rehabilitation (IPR): received vs no.

CIQ-R: Community Integration Questionnaire-Revised, ClinFIT: Clinical Functioning Information Tool, CRC: colorectal cancer, DASS: Depression Anxiety Stress Scale, EQ-5D-5L: Euro Quality of Life Scale; FACT-G: Functional Assessment of Cancer Therapy – General, FACT-C: Functional Assessment of Cancer Therapy – Colorectal Cancer. Values significant at 0.05 level are bolded.

- *Age*. Older participants exhibited significantly lower levels of functioning (ClinFIT), compared with younger participants. Additionally, older individuals reported poorer community integration, reflected by lower scores on the CIQ-R.
- *Gender*. Female participants had lower community integration (CIQ-R) scores and poorer QoL (EQ-5D-5L). This suggests potential disparities in post-treatment recovery and social reintegration between male and female survivors.
- *Fatigue and pain*. Participants experiencing ongoing symptoms of fatigue and pain reported poorer outcomes across multiple domains, with lower functioning scores (ClinFIT), reduced mental well-being (DASS), and diminished QoL (EQ-5D-5L, FACT-C). This underscores the significant impact of persistent physical symptoms on overall recovery and well-being.
- *Time since diagnosis*. A longer time since diagnosis was negatively associated with anxiety and stress levels (DASS-21 scores). Participants further along in their cancer trajectory also reported slightly better QoL outcomes on the EQ-5D-5L scale. These findings suggest a potential adaptive process over time, which warrants further investigation.
- *CRC type.* Survivors with colon cancer demonstrated slightly lower functional scores (ClinFIT G7) and higher levels of colorectal cancer-specific concerns (FACT-C) compared with their rectal cancer counterparts. This suggests that colon cancer survivors may experience more challenges in their overall functional capacity and may face more persistent concerns or difficulties related to diagnosis and treatment outcomes.
- *Stoma.* The presence of a stoma was negatively associated with overall mental health, as indicated by higher DASS-21 Total scores. However, it did not significantly impact other outcome measures. Additionally, no significant differences were observed between patients with a current stoma (either a non-reversed ileostomy or colostomy) and those who had a temporary, reversed ileostomy in any of the evaluated outcome measures.
- *Radiotherapy*. Receipt of radiotherapy was consistently associated with poorer outcomes across multiple domains, with lower scores for function (ClinFIT), psychological well-being (DASS-21 and FACT-C), community integration (CIQ-R), and QoL (EQ-5D-5L and FACT-C).

Other variables included in the multivariate regression models demonstrated minimal or no significant impact on the outcome measures. While these variables were statistically accounted for, their associations with the assessed scales were either negligible or not significant. This indicates that their contribution to explaining the variance in outcomes was limited compared with the primary factors identified (such as age, gender, symptom burden, and treatment history).

### Factors impacting current QoL

The logistic regression analysis revealed several significant predictors of poorer QoL based on the FACT-C total score (Table VI). The full model containing all predictors was statistically significant (χ² = 54.5, *p* < 0.001), indicating that it successfully distinguished between participants who reported poorer QoL and those who did not. Younger age (*p* = 0.017, OR = 0.013, 95% CI: 0.00–0.46), marital status (being married; *p* = 0.007, OR = 0.166, 95% CI: 0.05–0.62), lower pain levels (*p* < 0.001, OR = 0.10, 95% CI: 0.03–0.37), and lower fatigue levels (*p* = 0.003, OR = 0.017, 95% CI: 0.001–0.26) were associated with a reduced likelihood of reporting poorer QoL. No significant associations were identified for other variables, including sex, employment status, CRC type and duration, presence of a stoma, and prior chemotherapy or radiotherapy (*p* > 0.05). These findings suggest that older individuals, married participants, and those experiencing lower pain and fatigue levels were less likely to report poorer QoL.

**Table VI:** Logistic regression analysis of factors associated with poorer quality of life (FACT-C Total Score)^

Variables^§^	B	S.E.	Wald	*p*-value	OR	95% CI
Age	–4.315	1.800	5.743	**0.017**	0.01	0.00–0.46
Sex	–0.342	0.662	0.266	0.606	0.71	0.19–2.60
Marital status	–1.796	0.671	7.167	**0.007**	0.17	0.05–0.62
Employment	–1.289	0.749	2.960	0.085	0.28	0.06–1.20
CRC type	0.624	0.958	0.424	0.515	1.87	0.29–12.2
CRC duration	–0.267	0.736	0.131	0.717	0.77	0.18 -3.24
Stoma	0.662	0.872	0.577	0.447	1.94	0.35–10.7
CT	–0.238	0.878	0.073	0.787	0.79	0.14–4.41
RT	–0.596	0.976	0.373	0.541	0.55	0.08–3.73
Pain	–2.331	0.680	11.739	**< 0.001**	0.10	0.03–0.37
Fatigue	–4.096	1.396	8.615	**0.003**	0.02	0.001–0.26
IPR	–1.035	0.768	1.818	0.178	0.36	0.08–1.60

^ FACT-C total dichotomized using *a priori* cut-off of sample mean: ≤97.4: good QoL.

§ Variables references: Gender: male vs female; Age groups: ≤ 60 vs > 60 years; Marital status: married vs single; Employment status: employed vs unemployed; CRC type: colon vs rectal; CRC duration: ≤ 3 vs > 3 years; Stoma: none vs yes (present/reversed); Chemotherapy (CT): received vs none; Radiotherapy (RT): received vs none; Pain: present vs none; Fatigue: present vs none; Inpatient rehabilitation (IPR): received vs no.

CRC: colorectal cancer, CT: chemotherapy, FACT-C: Functional Assessment of Cancer Therapy – Colorectal cancer; OR: odds ratio, 95% CI: 95% confidence interval. Values significant at 0.05 level are bolded.

## DISCUSSION

This study evaluated factors influencing functioning, participation, and QoL in persons with CRC residing in the Australian community. It also sought information “needs” from the patient perspective, and patterns of care of CRC patients in the post-treatment phase. The findings highlight the complex interplay of sociodemographic, clinical, and treatment-related variables that shape the survivorship experience.

The sample characteristics reflect the typical demographic and clinical profile of CRC survivors, with a slight predominance of older, male, Caucasian, and a high prevalence of comorbidities (11). These findings align with the established epidemiology of CRC and emphasize the importance of considering coexisting health issues (23, 24). Consistent with other published reports, more than half of the participants reported advanced-stage CRC at diagnosis, which likely contributed to the high prevalence of ongoing symptoms, including fatigue, pain, and bowel dysfunction, as well as the observed socioeconomic and functional challenges (25–27).

Participants reported low ClinFIT scores, which suggest that many survivors retained basic physical functioning, a reflection of the benefits of modern cancer treatment modalities. This aligns with findings from other cancer cohorts, such as non-Hodgkin’s lymphoma, studied within the same institution (28). However, high rates of fatigue, pain, and bowel dysfunction highlight the lingering impact of CRC and its treatments on survivors’ daily lives. Psychological well-being scores, as measured by the DASS-21 (M ± SD = 11.3 ± 17.2), were relatively lower than normative data (18.4 ± 18.8) suggesting that the emotional burden of CRC, while present, was not overwhelming for most participants (18, 29). Nonetheless, ongoing fears of recurrence and other existential concerns were commonly reported, underscoring the need for continued psychological support in survivorship care (23, 30).

A decline in employment and marital/partnership status highlighted the socioeconomic impact of CRC, which limits reintegration into the community. The significant reduction in full-time employment reflects the long-term disruptions to occupational roles caused by CRC (and treatment), also noted in prior research (25, 26). Despite these challenges, many participants demonstrated resilience, as evidenced by positive community reintegration scores. Compared with the normative Australian sample (21), the CIQ-R total and subscales score in this study cohort is notably lower (M ± SD, normative sample vs study cohort): Total = 22.3 ± 4.7 vs 18.4 ± 6.5, Home integration = 7.7 ± 2.7 vs 6.9 ± 3.4; Social integration = 6.7 ± 2.0 vs 5.7 ± 2.0; Productivity = 4.7 ± 1.8 vs 3.0 ± 1.6; Electronic social networking = 3.2 ± 1.6 vs 2.9 ± 1.6). These findings show substantial challenges faced by CRC survivors in reintegrating into the community, at home, social, and productivity domains, and have limited use of electronic social networking platforms. This is attributable to the long-term physical, emotional, and social challenges associated with cancer survivorship, including fatigue, functional impairment, and changes in work and social roles. Tailored interventions focusing on improving social and occupational participation and enhancing independence in daily living could help address these gaps in community integration.

The study findings highlight a substantial burden of “unmet” supportive care needs, particularly in the domains of existential survivorship and comprehensive cancer care. Common unmet needs included stress management, access to complementary therapies, support for side effects, and body image adjustment. Existential survivorship challenges, such as fear of recurrence, uncertainty about the future, and role adjustment, had both high met and unmet needs, aligning with the findings of previous studies (23, 24, 31). Participants reported higher met needs in the “Comprehensive Cancer Care” and “Information” domains, reflecting effective communication and resource availability in the tertiary setting, which were comparable to the data for overall cancers (23, 31). However, “unmet” needs persisted for specific types of information, such as managing long-term side effects. Interestingly, the “Relationship” domain had the lowest levels of met and unmet needs indicating stable social relationships, which is consistent with other reports (23, 31). Further, the QoL domain had moderate levels of “unmet” and “met” needs, which are comparable to data from other reports (23, 31). Overall, while many needs are being met, higher unmet needs in existential survivorship and QoL domains highlight the need for targeted interventions to address these challenges.

### Factors influencing functioning and QoL

This study found that younger age, being married, and experiencing lower levels of pain and fatigue were significantly associated with a reduced likelihood of reporting poorer QoL. In contrast, factors such as sex, employment status, CRC type and duration, presence of a stoma, prior chemotherapy or radiotherapy, and inpatient rehabilitation were not significantly associated with QoL outcomes. Overall, the reported QoL outcomes were surprisingly positive, with FACT-C and EQ-5D-5L scores indicating good health and well-being, reflecting a favourable long-term recovery trajectory. The potential explanation for these findings lies in the characteristics of the study cohort. As most participants (55%) had low GI (rectal cancers), these inherently involve less invasive surgery (typically more localized) with fewer postoperative complications and shorter recovery periods compared with other GI cancers. Further, older age was also associated with poorer physical functioning and community integration, while female participants reported lower community reintegration scores. These findings suggest age- and gender-specific challenges in CRC survivorship, specifically in terms of reduced physical functioning and societal participation. Dunn et al. in a population-based longitudinal study (*n* = 1,966) reported similar findings, suggesting later-stage disease and female gender, lower optimism, poorer social support, negative cognitive appraisal, and younger age were associated with poorer health-related QoL (12). Other literature identifies older adults and women as vulnerable groups requiring targeted interventions (9, 12, 32). Consistent with previous studies, fatigue and pain emerged as critical determinants of poorer outcomes across multiple domains, including physical functioning, mental well-being, and QoL, emphasizing the need for effective symptom management strategies (33, 34). Paul et al. in a longitudinal study (*n* = 539) reported an increase in various CRC-related issues, specifically pain and discomfort (11). These findings highlight the importance of social support and effective symptom management in sustaining better QoL in CRC survivors. Targeted interventions addressing pain, fatigue, and psychosocial factors may further enhance overall well-being in this population.

A longer time since diagnosis was associated with lower anxiety and stress levels, suggesting an adaptive process over time. However, the persistence of unmet needs and functional impairments underscores the importance of ongoing support throughout the survivorship trajectory. Participants who received radiotherapy reported poorer outcomes in functioning, psychological well-being, community integration, and QoL, highlighting the need for proactive side-effect management (35). Despite these challenges and ongoing issues such as fear of recurrence, sleep disturbances, fatigue, and pain, most participants reported good psychological well-being, satisfaction, and adjustment. This aligns with evidence suggesting many CRC survivors adapt well, particularly with adequate support (36, 37). One possible explanation for this is the “response shift” phenomenon where individuals recalibrate their internal standards, values, or QoL perceptions following health changes (38). Patients may reassess their limitations in daily living, reset their goals, and perceive the impact of their condition as less significant than initially thought (39). While this phenomenon is well documented and is acknowledged by clinicians, its clinical significance and broader implications remain unclear (28, 38, 39). Further research is needed to understand response shifts and address unmet psychological, social, and informational needs in survivorship care.

### Study limitations

This study has several limitations. This is a descriptive-analytical study without a control group, limiting the ability to establish causal relationships. The study cohort consisted of a small and selective group of participants, who agreed to participate, from a single tertiary metropolitan institute listed in a CRC database, potentially restricting the generalizability of the findings to other settings or populations. However, the cohort included all CRC episodes for 5 years and covered a wide geographical area, making it broadly representative of CRC patients in the community. Additionally, the demographic and clinical characteristics of participants are comparable to those reported in other CRC cohorts. Our study cohort comprised a higher proportion of rectal cancer patients than typically observed in existing literature. This overrepresentation may be attributed to a potential selection bias, where rectal cancer patients showed greater willingness to participate in the study. However, investigating the specific reasons for this disproportionate representation was beyond the scope of our current research. Several potentially influential factors, such as pre-diagnosis functional and cognitive levels, socioeconomic status, and cultural considerations, were not evaluated in this study, as these were beyond the scope. Further, this study did not employ a defined minimum post-discharge interval as part of the inclusion criteria, which may have limited the comparability of participants’ community reintegration experiences. Establishing such a criterion could improve consistency in future research and is an important consideration for study design. Despite employing a comprehensive set of validated tools to assess functioning, QoL, and community reintegration, issues outside the domains of these measures could not be captured. To minimize recall bias, all outcomes were limited to the participants’ current status, and clinical and demographic data were cross-verified using electronic medical records. The large number of univariate and multivariate regression analyses performed also warrant caution. This approach aligns with the study’s exploratory nature and the cross-sectional design, which prevents any inference of causality. Potential confounders and interaction effects were not explored in depth, and no correction for multiple comparisons was applied, which may increase the risk of Type I error.

### Clinical and research implications

The significant impact of fatigue, pain, and other persistent symptoms on functioning and QoL can be mitigated by integrating symptom-focused interventions (e.g., fatigue management, pain control strategies, pelvic floor rehabilitation, etc.) into standard follow-up care. The disparities observed in community reintegration and QoL among older adults and female survivors highlight the need for tailored support programmes that address age- and gender-specific barriers to recovery. Additionally, the long-term negative effects of radiotherapy on physical, psychological, and social well-being emphasize the importance of proactive side-effect management and rehabilitation. Further, the high prevalence of unmet supportive care needs, particularly in existential survivorship and comprehensive cancer care, calls for a more holistic, interdisciplinary approach to survivorship care to enhance recovery and resilience, and improve overall survivorship experiences for individuals living with and beyond CRC.

Future research should explore the mechanisms underlying the observed disparities in outcomes and evaluate the effectiveness of tailored interventions in improving QoL and functional recovery. Longitudinal studies with larger, more diverse cohorts and investigations of pre-diagnosis and care process factors will improve understanding of their influence on CRC survivorship/trajectories and changes in survivorship experiences over time to promote resilience and positive community adjustment.

### Conclusion

This study underscores the need for personalized, interdisciplinary survivorship care throughout the disease continuum to address the diverse needs of CRC survivors in the community. Targeted programmes for older adults, women, and those following radiotherapy will address the unmet survivorship needs. We envisage that the findings will assist in health service management planning, knowledge transfer amongst healthcare professionals, mapping effective resource allocation to patients’ needs, and delivery of effective, sustainable care for improved clinical outcomes.
